# The Prospects of Using Structural Phase Analysis of Microcalcifications in Breast Cancer Diagnostics

**DOI:** 10.3390/diagnostics13040737

**Published:** 2023-02-15

**Authors:** Artem Piddubnyi, Olena Kolomiiets, Sergey Danilchenko, Andriy Stepanenko, Yuliia Moskalenko, Roman Moskalenko

**Affiliations:** 1 Department of Pathology, Sumy State University, 40022 Sumy, Ukraine; 2 Ukrainian-Swedish Research Center SUMEYA, Sumy State University, 40022 Sumy, Ukraine; 3 Institute of Applied Physics, NAS of Ukraine, 40007 Sumy, Ukraine; 4 Department of Electronics, General and Applied Physics, Sumy State University, 40007 Sumy, Ukraine; 5 Department of Oncology and Radiology, Sumy State University, 40022 Sumy, Ukraine

**Keywords:** breast cancer, microcalcification, phase analysis

## Abstract

The detection of microcalcifications in the breast by mammography is of great importance for the early diagnostics of breast cancer. This study aimed to establish the basic morphological and crystal-chemical properties of microscopic calcifications and their impact on breast cancer tissue. During the retrospective study, 55 out of 469 breast cancer samples had microcalcifications. The expression of the estrogen and progesterone receptors and Her2-neu showed no significant difference from the non-calcified samples. An in-depth study of 60 tumor samples revealed a higher expression of osteopontin in the calcified breast cancer samples (*p* ˂ 0.01). The mineral deposits had a hydroxyapatite composition. Within the group of calcified breast cancer samples, we detected six cases of colocalization of oxalate microcalcifications together with biominerals of the usual “hydroxyapatite” phase composition. The simultaneous presence of calcium oxalate and hydroxyapatite was accompanied by a different spatial localization of microcalcifications. Thus, the phase compositions of microcalcifications could not be used as criteria for the differential diagnostics of breast tumors.

## 1. Introduction

Breast cancer (BC) is the most common cancer in women worldwide and causes the highest mortality. In the United States, BC is the most common cancer type, and in 2021, cases of BC constituted 14.8% of all malignancies [1].

However, improved treatment and early diagnostics have increased the survival rates of BC patients [2]. Early BC diagnostics has the highest priority worldwide, since the 5-year survival rate for stage I BC is about 100%, and for stage IV it does not exceed 20% [3]. A key achievement for early diagnostics is the introduction of mammography screening programs for women in high-risk groups [4]. Microcalcifications are the most important signs of BC in mammography. Pathological biomineral deposits are markers of the type of the breast pathology present and indicate the stage of the disease [5]. AI-based technologies are promising tools for effective diagnosis as well as prognosis assessment [6].

BC microcalcifications are usually associated with degenerative necrotic changes in tumor tissue [7]. The presence of microcalcifications in the breast correlates with a worse prognosis, especially because of the higher incidence of lymph node invasion and rapid metastasis [8]. 

Breast microcalcifications mainly consist of calcium oxalate (calcium oxalate in the crystalline form of weddellite) or calcium phosphate with an apatite structure. The presence of tricalcium magnesium phosphate with the structure of magnesium whitlockite (magnesium whitlockite) has also been shown [9,10]. It is believed that the presence of calcium oxalate is associated with benign breast tumors, whereas the detection of calcium phosphates points to possible breast malignancy [9,11,12]. The phase composition of BC microcalcifications obviously depends on the difference in the biochemical processes of benign and malignant tumors. Microcalcifications can also lead to secondary damage of tumor tissue and further distort its metabolism [13].

The simultaneous presence of calcium oxalate and apatite in breast tumor tissue has not been considered. Obviously, this is due to attempts to emphasize the diagnostic perspective of structural phase analysis in BC diagnostics, but does not fully correspond to the reality. The mechanisms of the transformation of BC microcalcifications in the case of tumor progression remain unclear.

This study aims to establish the basic morphological and crystal-chemical properties of microscopic calcifications and their influence on breast cancer tissue.

## 2. Materials and Methods

### 2.1. The Ethics Committee 

The study was approved by the ethics committee of the Medical Institute of Sumy State University (Proceedings 3/03, 9 February 2021).

### 2.2. Retrospective Study 

We run a retrospective analysis of 469 invasive BC biopsy samples from the archives of the Department of Pathology of Sumy State University for the period 2014–2019. Samples were evaluated for the presence of calcifications and for the immunohistochemical expression of Estrogen (ER) and Progesterone (PR) receptors and Human epidermal growth factor receptor 2 (Her2-neu). 

### 2.3. Samples Collection

Tissue samples were collected at the Sumy Regional Clinical Oncology Center (Sumy, Ukraine). After histological examination, 30 samples of BC tissue with biomineralization (Group I, Supplementary Table S1) and 30 non-mineralized samples (Group II, Supplementary Table S2) were selected for this study. 

### 2.4. Histopathology Examination

The tissue was fixed in a 10% neutral (buffered) formaldehyde solution for 24 h. All tissue processing procedures (fixation, paraffin saturation, embedding) were performed according to the generally accepted methods. Serial sections with a thickness of 4–5 μm were stained with Mayer’s hematoxylin and eosin. 

### 2.5. Histochemistry Examination

The von Koss staining was used to verify the presence of calcium phosphate in BC biominerals. The rehydrated histological sections were treated with a 5% aqueous solution of silver nitrate under the direct light of a 60 W lamp for 60 min. Subsequently, the tissue samples were treated with sodium thiosulfate (5% aqueous solution). Nuclei were counterstained with an aqueous solution of nuclear fast red (1:1000).

The Pierce method of alizarin red staining revealed the calcium depositions (pH correction to 6.3–6.5 with NH_4_OH). Further, sections were washed with distilled water for 10 s and treated with a solution of acidified ethanol for 15 s.

### 2.6. Immunohistochemistry (IHC) of BC Tissue

Serial sections with a thickness of 4 μm were applied to the SuperFrost adhesive slides (Thermo Scientific, Waltham, MA, USA). The deparaffined sections were subjected to the antigen unmasking by the heat treatment in 0.1 M citrate buffer (pH 6.0) at of 95–98 °C. The “UltraVision Quanto Detection System HRP Polymer” (Thermo Scientific, Waltham, MA, USA) was used to visualize the IHC. Endogenous peroxidase activity was blocked by 3% hydrogen peroxide. “Ultra V Block” was used to prevent non-specific reactions and background staining. Reaction was amplified with “Primary Antibody Amplifier Quanto”. Diaminobenzidine (DAB) was used as a chromogen. The nuclei were counterstained with Mayer’s hematoxylin. Primary antibodies against osteopontin (OPN) (Abcam, ab-8448, dilution 1:300) were used. In general, the procedure of immunohistochemical staining of tissue was performed according to the previously described method [14]. At least six different fields of view (FOV) with diameter of 1 mm were analyzed for each sample. The IHC results were presented as a mean number of OPN-positive cells per FOV.

### 2.7. Scanning Electron Microscopy with Energy Dispersive Spectroscopy (SEM-EDS)

Histological sections with a thickness of 7–10 μm were placed on graphite blocks. Samples were preheated in a thermostat at a temperature of 60 °C for 30 min. After that, paraffin sections were deparaffinized in xylene (3 portions for 5 min) and rehydrated in 96% ethanol (3 portions for 5 min) and distilled water. This also removed the need for carbon coating. We used an SEO-SEM Inspect S50-B (SEO, Sumy, Ukraine) scanning microscope with energy dispersion spectrometer AZtecOne with detector X-MaxN20 (Oxford Instruments plc, Oxford, UK). EDX spectra were analyzed with standard software of the microanalysis system. The mineral composition of microcalcifications was identified based on the elemental composition (Ca/P ratio).

### 2.8. Transmission Electron Microscopy with Electron Diffraction (TEM-ED)

TEM-ED was performed with TEM-125K (SELMI, Sumy, Ukraine) for the study of the morphological features and sizes of the nanocrystals of the deposits. The powder of mineralized tissue was sonicated in distilled water with UZDN-A (SELMI, Sumy, Ukraine). The specific power was 15–20 W/cm^2^ at an emitter frequency of 22 kHz. The suspension (few drops) was applied to a vertical upward ultrasonic emitter UZDN-A and sprayed for 2–3 s at the optimal power. The sprayed aerosol was mounted on a thin carbon substrate film (15–25 nm) on a copper mesh of the sample holder. ED images and microphotographs were captured at accelerating voltage U_acceleration_= 90 kV.

### 2.9. Statistical Analysis

The normality of all datasets was assessed by the Shapiro–Wilk test. Student’s *t*-test was applied for data analysis with a normal distribution. The Mann–Whitney U-test was applied for nonparametric datasets. The results were considered statistically significant with a probability of more than 95% (*p* < 0.05). To compare the level of the Her2-neu expression, the Chi-square test was used. Statistical analysis was performed in Microsoft Office Excel 2016 with the addon AtteStat (version 12.0.5). All graphs were made with GraphPad Prism 9.0.

## 3. Results

### 3.1. A Retrospective Study of the ER, PR and Her2-neu Expression in BC Tissue with Calcification

**Figure 1:**
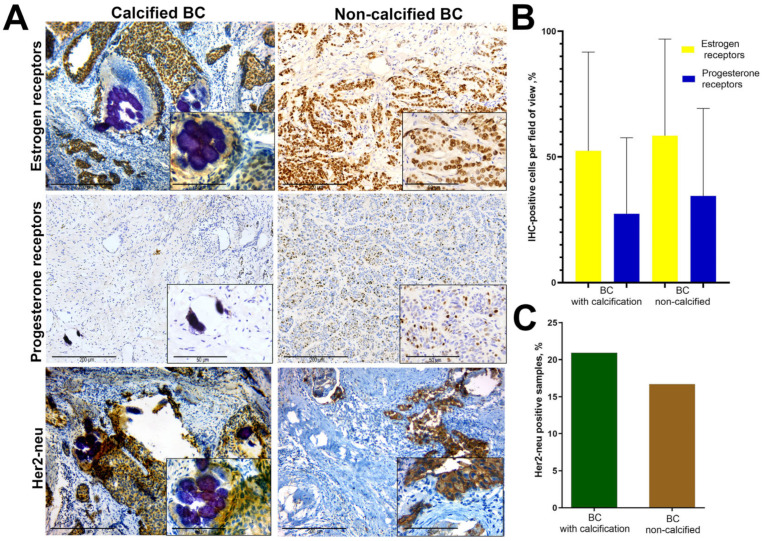
Retrospective IHC study of BC biopsies. (**A**)—IHC detection of ER, PR and Her2-neu in the BC tissue; (**B**)—levels of IHC expression of hormone receptors in the BC tissue, mean values; (**C**)—IHC expression of Her2-neu.

In this study, we examined 469 BC biopsy samples. At the initial stage, each sample was examined by hematoxylin-eosin staining. In 55 samples (11.73%) the presence of biominerals was detected, whereas 414 samples (88.27%) showed no signs of calcification. Calcified samples had 52.45 ± 5.3% ER positive and 27.36 ± 4.08% PR positive BC cells. Non-calcified samples of BC had 58.63 ± 4.25% (*p* > 0.05) and 34.57 ± 4.74% (*p* > 0.05) ER and PR positive cells, respectively (Figure 1B).

IHC expression of Her2-neu protein was manifested by distinct membrane staining (Figure 1A); cytoplasmic staining of tumor cells was not evaluated. The assessment of BC Her2-neu status was performed according to the HercepTest system (complete membrane staining of more than 20% of tumor cells corresponds to positive status) [15]. We detected 20.93% Her2-neu positive calcified samples and 16.71% (*p* > 0.05) non-calcified samples (Figure 1C).

### 3.2. Macroscopic Study

Macroscopically, all tumors showed no significant visual differences. BC was detected as a well-defined, dense nodule with a different color and consistency from the surrounding tissues. Most of the samples had a gray color with yellow areas of necrosis and hemorrhages, and they were tightly connected to the surrounding tissues. The tumors usually had jagged edges and very dense consistency. The grossing of some samples of group I was difficult because of the presence of solid dense inclusions. 

### 3.3. Histopathology

All the samples were defined as invasive ductal BC. The pathological tissue of both groups had highly similar pathomorphological structures. The main tumor mass was represented by polymorphic glands formed by atypical cells and surrounded by stroma. In some cases, cancer cells formed irregularly shaped solid structures. The amount of stroma in BC samples varied significantly, as did its maturity. In some BC samples, we noticed the presence of intraductal small polymorphic cells which formed papillary, cribrous and solid structures. Tumor cells contained polymorphic and hyperchromic nuclei with coarse chromatin and mitotic figures.

**Figure 2:**
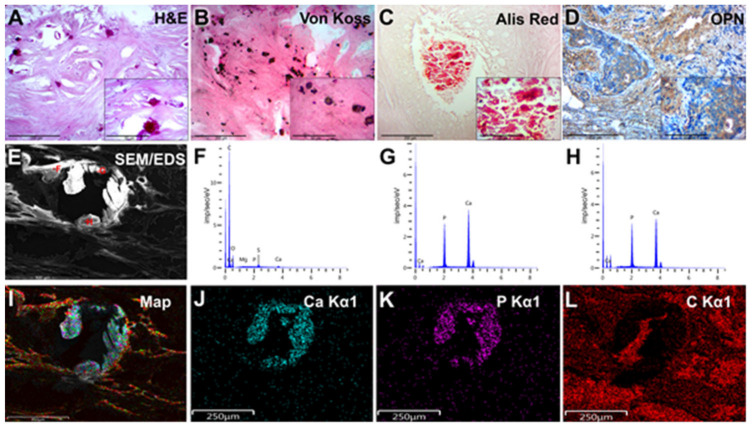
Examination of BC tissue with calcifications (group I). (**A**)—hematoxylin-eosin staining; (**B**)—von Koss staining of BC tissue; (**C**)—alizarin red staining of BC tissue. (**D**)—IHC expression of OPN. The figures in the inserts correspond to the enlarged area of this sample. (**E**)—SEM of BC. (**F**–**H**)—EDX spectra correspond to the measurement points in Figure E. (**I**)—EDX mapping of BC tissue: turquoise corresponds to calcium ions (**J**); purple—phosphorus (**K**); red—carbon (**L**). Magnification is indicated in the lower left corner of the image as a marker.

The presence of microcalcifications was the main selection criterion for group I. Biomineral deposits had the form of large granules or rounded bodies, mainly as white opaque concretes with round or irregular shapes (Figure 2A). These biomineral deposits became visible by von Koss silver staining (Figure 2B). Biomineral deposits were detected among tumor masses and in the ducts, and necrotic areas and had a tendency to group (Figure 2C). 

During the sectioning and staining, a significant number of microcalcifications were mechanically damaged (destruction/damage with a microtome knife). We have reported similar phenomena in our previous publication on microcalcifications [16].

**Figure 3:**
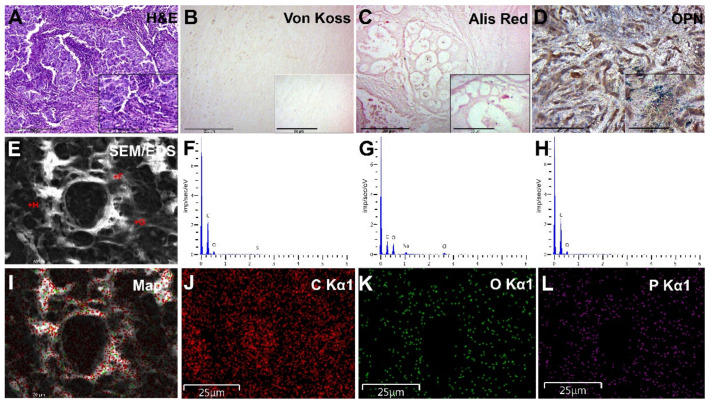
Examination of BC tissue without calcifications (group II). (**A**)—hematoxylin-eosin staining; (**B**)—von Koss staining of BC tissue; (**C**)—alizarin red staining of BC tissue; (**D**)—IHC expression of OPN. The figures in the inserts correspond to the enlarged area of this sample. (**E**)—SEM of BC. (**F**–**H**)—EDX spectra correspond to the measurement points in Figure E. (**I**)—EDX mapping of BC tissue: red corresponds to carbon ions (**J**); green—oxygen (**K**); purple—phosphorus (**L**). Magnification is indicated in the lower left corner of the image as a marker.

Group II samples consisted of atypical glands surrounded by a tumor stroma. We noted solid and intraductal tumor growth of BC (Figure 3A). Histochemical von Koss and alizarin red staining revealed no signs of calcification (Figure 3B,C).

### 3.4. Immunohistochemistry

We detected the intensive expression of the OPN in fibroblasts, macrophages and, particularly, tumor cells (Figure 2D). We also pointed out the association of OPN overexpression with the presence of microcalcifications. The high background staining by chromogen (DAB) was observed and was related to the deposition of significant amounts of small calcium phosphate and biomineral fragments on the tissues during IHC.

OPN IHC staining of group II samples revealed a moderate accumulation of this protein in the tumor microenvironment and tumor tissue (Figure 3D). We noted a moderate cytoplasmic expression of OPN in fibroblasts, macrophages, and tumor cells. In this group of samples, the background staining was lower.

### 3.5. Scanning Electron Microscopy

During the SEM, biominerals were detected as bright, white–gray rounded particles, or their fragments and plates localized among the tumor masses or their stromal components (Figure 2E). Large biomineral formations had signs of destruction and fragmentation that occurred due to sample damage during the sectioning. There were no signs of close biomineral incrustation into the histological structures of BC tissue.

### 3.6. EDX Analysis of BC Microcalcifications

According to the element distribution maps and the Ca/P ratio from the EDX spectra at selected points (Figure 2E–H), the deposited mineral was considered calcium phosphate with an apatite composition. Obviously, the result of the F point was obtained from the organic component of the BC sample. The carbon distribution had no visible signs of deposition at the sites of mineralization. This indicates the shielding of the carbon table-substrate by the calcified tissues. The Ca/P ratio (obtained by the EDX analysis) (Figure 2G–H) was more consistent with calcium hydroxyapatite (HA) than with tricalcium phosphate (TCP), even without considering possible substitutions of Ca for Mg and Na in the apatite lattice. All analyzed points showed a high level of mineralization, except for point F, where a low Ca/P ratio and high levels of carbon, oxygen and sulfur indicated the presence of a significant amount of organic matter.

We detected no signs of biomineral deposition in any of the group II BC samples (Figure 3E–L). 

### 3.7. Transmission Electron Microscopy with Electron Diffraction

**Figure 4:**
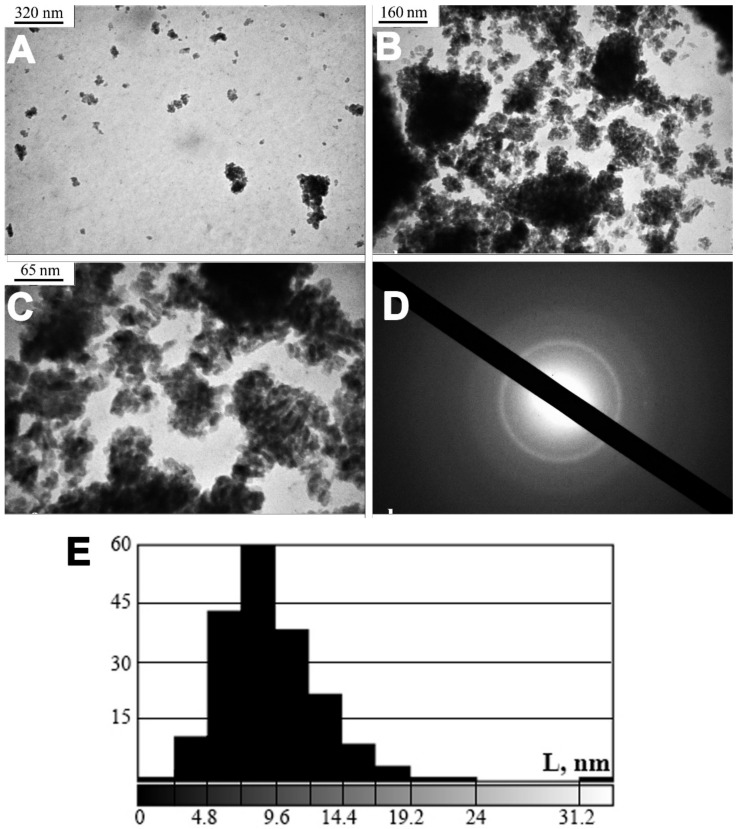
TEM with ED of calcified BC samples. (**A**–**C**)—crystals; (**D**)—electron microdiffraction; (**E**)—histogram of the crystal particle size distribution. *X*-axis—nanocrystal size; *Y*-axis—numbers of samples.

The microstructures of BC calcifications and the corresponding electrogram are shown in Figure 4A–C. TEM and ED revealed that samples were single-phase and had a polycrystalline structure. Individual monocrystalline particles of BC calcifications had sizes ranging from 3 nm to 20 nm, the most predominant size being 9 nm (Figure 4E). Nanocrystals of BC calcifications were transformed into polycrystalline and mechanically stable agglomerations with sizes of 100–300 nm. There was no recrystallization with a marked increase in the size of the individual components.

### 3.8. The Problem of Oxalates

Among the group I samples, we detected six cases of a specific type of microcalcifications phase composition. They were detected together with microcalcifications of the usual “hydroxyapatite” phase composition. This may be potentially important in the development of a method for the differential diagnosis of BC. Therefore, we identified these samples in a separate section of this study.

**Figure 5:**
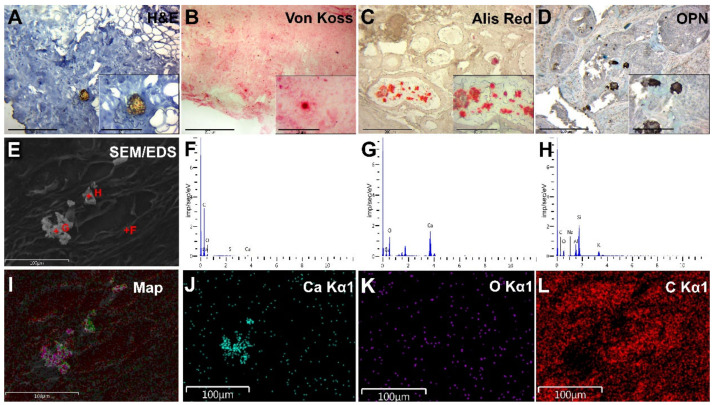
Examination of BC tissue with calcifications of non-hydroxyapatite origin (group II). (**A**)—hematoxylin-eosin staining; (**B**)—von Koss staining of BC tissue; (**C**)—alizarin red staining of BC tissue. (**D**)—IHC expression of OPN. The figures in the inserts correspond to the enlarged area of this sample. (**E**)—SEM of BC. (**F**–**H**)—EDX spectra correspond to the measurement points in Figure E. (**I**)—EDX mapping of BC tissue: turquoise corresponds to calcium ions (**J**); purple—phosphorus (**K**); red—carbon (**L**). Magnification is indicated in the lower left corner of the image as a marker.

We detected amber-colored biomineral deposits in the adjacent intact tissue of group I tumor samples. They were more transparent and had a complex polygonal structure (Figure 5A). In general, the structure of microcalcifications of this type was similar to “breadcrumbs” or lumps with small elements. These structures were located in the lumen of the dilated glands or were associated with their lumen (located near the glands). It is important to note that the glands that contained microcalcifications had a normal histological structure.

Histochemical von Koss staining provided conflicting results. Despite the absence of the classical brown color (precipitation of silver on calcium phosphate compounds), we found reddish staining of the biomineral deposits (Figure 5B). This may be due to the precipitation of a counterstaining dye (nuclear fast red) to the biomineral structures. On the other hand, these intraluminal calcified structures had an unusual alizarin red staining (Figure 5C). 

During the IHC detection of the OPN expression, we noted its moderate expression in tumor microenvironment and tissue, which was similar to that of other samples of groups I and II. However, the accumulation of this protein on the surfaces of microcalcifications appeared more like an artifact, because it captured only the boundaries of the crystal structure, and did not accumulate on its entire cross-section (Figure 5D). The background staining was low.

The SEM showed that mineral deposits had the structures of irregularly shaped particles (30–40 μm) with granulation and growths that were not consolidated with the surrounding/adjacent non-neoplastic breast tissue (Figure 5E). Small growths on microcalcifications were identified as crystals. According to the maps of the distribution of the elements (points are indicated in Figure 5E), the most likely contender was calcium oxalate. This result was confirmed by the EDX spectra and mapping (Figure 5F–L). To some extent, calcified particles may be mineral–organic composite formations, as evidenced by the EDX spectrum at F point (Figure 5F). The EDX spectrum marked H indicates the possible formation of mineral particles that did not contain calcium.

**Figure 6:**
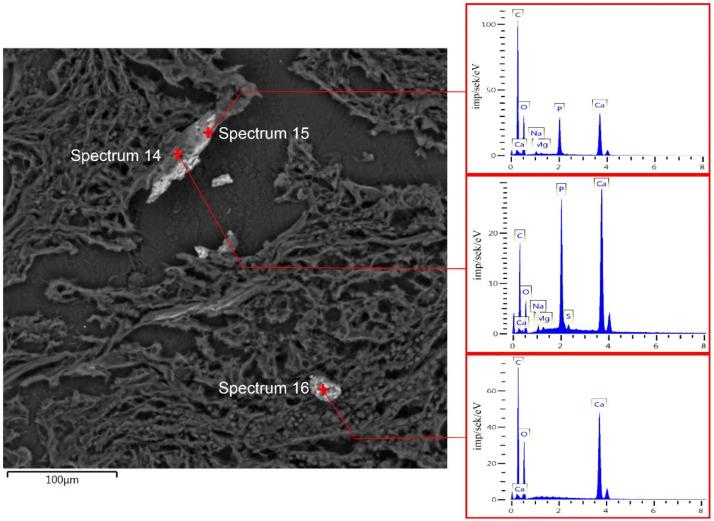
SEM and EDX of BC with microcalcifications. The spectra correspond to the measurement points in the figure. Spectra 14 and 15—hydroxyapatite; spectrum 16—calcium oxalate.

It should be noted the simultaneous detection of microcalcifications of apatite and oxalate nature in BC samples (Figure 6). We detected the fragmented calcification in the tumor tissue. The EDX spectra 14 and 15 indicate its hydroxyapatite structure. Spectrum 16 of the separate biomineral (most likely dislocated from adjacent tissue) showed its oxalate origin.

## 4. Discussion

The diagnostic value of breast microcalcifications has been in the spotlight of scientists in recent years. BC microcalcifications were first identified as an important diagnostic marker of malignant tumors in 1951 [17]. In the fifth edition of the standardized system for the assessment of breast pathology by mammography, ultrasound and MRI (BI-RADS) (The Breast Imaging Reporting and Data System), microcalcifications have a separate section [6]. They have an important value for the diagnostics of BC at the initial stages [18,19]. Of course, the very presence of microcalcifications provides only general information, because they can be detected in both malignant and benign breast tumors.

The chemical and phase composition of BC microcalcifications has been actively studied in recent decades. It has been found that the main crystalline phases of BC microcalcifications are calcium oxalate (type I calcification) and calcium hydroxyapatite (type II calcification) [20,21,22]. Calcium oxalate is produced by the apocrine cells of the breast [23]. They are mainly associated with benign cystic changes, but may also be associated with BC. Calcium oxalate cannot be metabolized by mammalian cells, and there is new evidence that oxalate exposure can affect epithelial cells, causing cellular and genetic changes [23]. Cell oxalates induce changes in normal breast epithelial cells and promote the transformation of normal breast cells into tumor cells [24]. This coincides with the results of this study, because we found the presence of oxalate structures in areas of intact breast tissue. However, no oxalate calcifications were found in the tumor tissue. The presence of microcalcifications of oxalate origin in BC tissue can be explained by the development of malignant tumors on the background of pre-existing benign pathology, which is inherited by calcium oxalate microcalcifications. The simultaneous presence of hydroxyapatite and oxalate biominerals in the tissue of invasive BC complicates the use of crystal-phase differentiation of pathological biominerals for non-invasive radiological diagnostics.

Calcium phosphate is more easily recognized in histology and is more often associated with malignant breast neoplasms. Changes and variations in the levels and content of calcium carbonate in hydroxyapatite can affect the growth of BC cells [23]. It has been shown that BC with microcalcifications is more often associated with lymph node invasion [25], so these data suggest that breast microcalcifications worsen prognosis in BC patients [8]. However, this study does not support this idea. We found no significant differences in the incidence of lymph node metastases within BC with and without microcalcifications (15 and 13 samples respectively) (Supplementary Tables S1 and S2). 

Our study showed the presence of hydroxyapatite in all samples of BC with biomineralization (group I). However, we also detected six cases of microcalcifications with oxalate structures. Nevertheless, the fundamental difference in the localization of these formations should be noted again. The combination of microcalcifications of hydroxyapatite and oxalate phase composition in some cases of invasive BC reduces the diagnostic value of pathological biomineralization. However, the spatial localization of oxalate microcalcifications in our study always corresponded to areas of tissue without tumor growth. They were predominantly localized in the lumens and ducts of the dilated glands and connective and adipose tissue of the breast. Microcalcifications of a hydroxyapatite nature are easily detected by routine histopathological examination, as they turn dark blue by hematoxylin staining. Calcium oxalate microcalcifications have an amber–golden color. It has been shown that polarization microscopy could be used to detect calcium oxalate crystals [24]. At the same time, microcalcifications of oxalate structure have dubious von Koss and OPN staining. For hydroxyapatite, both methods are sensitive and provide unequivocally positive reactions. Both types of microcalcifications have positive staining with alizarin red. Based on this and previous studies, we compared the diagnostic features of both crystalline forms of microcalcification (Table 1). This information may be of practical value to clinicians, including pathologists.

The prognostic value of BC microcalcifications is also actively studied and discussed [9,11,19,21,23]. There are several studies that substantiate the association between BC microcalcifications and an unfavorable prognosis [8,11,16,20]. The estimation of IHC expression of ER, PR and Her2-neu is mandatory for patients with BC according to NCCN (National Comprehensive Cancer Network) standards to determine treatment tactics. For example, E.Y. Chae et al., (2016) reported an association between ER-positive status and the presence/absence of calcification with a worse prognosis for patients [26]. We revealed no statistically significant difference in the IHC expression of these markers in patients with and without microcalcifications. Recent studies have established that calcification is an independent prognostic factor of BC and does not correlate with IHC profile, tumor size or the presence of lymph node metastases [27]. However, there is an interesting opinion about the relationship between the formation of microcalcifications and so-called osteological diseases, such as osteoarthritis, rheumatoid arthritis, etc [28]. S. Wang et al., (2022) showed the presence of tumor cells and cells of the tumor microenvironment with an osteoblastic immunophenotype. Cells of the tumor microenvironment, particularly tumor-associated macrophages, can secrete BMP-2 and thus stimulate the formation of [29]. In our study, we found a similar propensity for the expression of the osteoblastic marker osteopontin by tumor cells and the BC microenvironment.

OPN is a small protein that is actively involved in the development and formation of bones and the processes of pathological vascular biomineralization. OPN also inhibits hydroxyapatite formation by binding to the surface of the crystals [30]. Given this functional feature, OPN can always be detected in the foci of calcification. This protein is a clear and early marker of biomineralization. OPN overexpression also promotes lymphatic invasion and its high expression associated with decreased chances of remission and overall survival [31,32].

**Figure 7:**
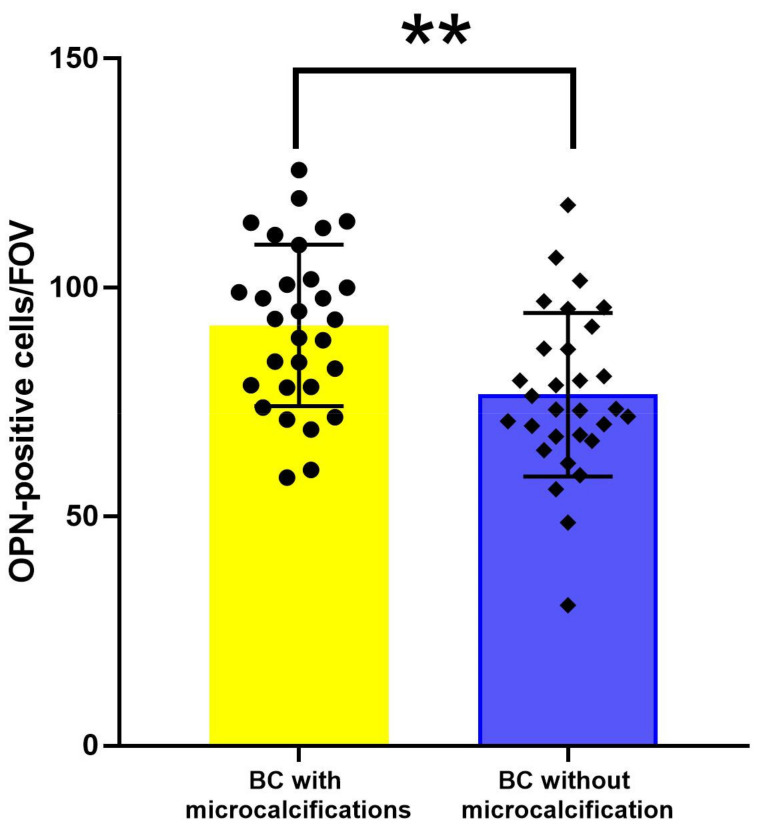
The evaluation of OPN expression in BC tissue. •, ▪—individual values from I and II group correspondingly, **—*p* < 0.01.

The average OPN expression in BC tissue with biomineralization (91.745 ± 3.22) is higher than in tumor tissue without calcification (76.62 ± 3.26) (*p* ˂ 0.01). The presence of pathological biomineralization leads to a high level of OPN protein expression in the tissue of BC (Figure 7).

OPN was detected in both the cytoplasm of cells (main producers of this protein) and extracellular structures and the surfaces of calcifications, fibers and sites of biomineral formation. In general, the IHC results coincided with numerous evidence of the direct involvement of OPN in the formation and development of pathological biomineralization [33]. In addition, OPN is a normal component of elastic fibers in the breast. It obviously causes a high level of IHC expression in the non-calcified samples. Theoretically, this will complicate the use of OPN as a diagnostic marker for BC. Nevertheless, the lower intensity of OPN expression in BC with oxalates points to its questionable role in the formation of these microcalcifications.

SEM with EDX confirmed that the biomineral part of BC microcalcifications consisted mainly of hydroxyapatites, which is also consistent with previous studies [10,22,34]. According to the Ca/P ratio (and to the presence of traces of organic matter on elemental mapping), microcalcifications of tumor tissue correspond to a mature mineral. The simultaneous presence of calcium oxalate and hydroxyapatite was accompanied by a different spatial localization of microcalcifications. It is obvious that the development of malignant breast tumors occurs against a background of pre-existing benign pathology. This newly developed cancer may inherit pre-existing calcium oxalate microcalcifications.

## 5. Conclusions

Our study showed that microcalcifications had no effect on the level of expression of estrogen receptors, progesterone receptors and epidermal growth factor 2 in breast cancer tissue. 

Higher levels of osteopontin immunohistochemical expression were detected in breast cancer tissue with microcalcifications. Due to its known protumorogenic effect, osteopontin has a potential negative prognostic value for the progression of breast cancer. However, the involvement of osteopontin in the carcinogenesis of breast cancer obviously affects the high level of its immunohistochemical expression in the non-calcified tissue.

According to the Ca/P ratio (and the presence of organic matter traces), microcalcifications of tumor tissue correspond to mature hydroxyapatite.

The simultaneous presence of calcium oxalate and hydroxyapatite was accompanied by a different spatial localization of microcalcifications. This might be related to the different conditions of their formation, and complicates the potential use of this phenomenon for the early diagnostics of breast cancer.

## Figures and Tables

**Table 1:** Differential diagnosis of BC microcalcifications.

Feature	Hydroxyapatite	Calcium Oxalate
Localization	Tumor masses, vessels, necrotic masses	The lumen of glands, detritus in the ducts
Hematoxylin-eosin staining	Dark blue	Amber
Polarization	−	+
Von Koss staining	+	Doubtful
Alizarin red staining	+	+
IHC OPN expression	+	Doubtful
Crystalline form	Hexagonal	Rectangular crystals, parallelepiped

## Data Availability

Data available within the article.

## Supplementary Materials

The following are available online at https://www.mdpi.com/article/10.3390/diagnostics13040737/s1. Table S1: Group I—The list of patients with breast cancer microcalcifications, Table S2: Group II—The list of patients with non-calcified breast cancer.
