# Supplementary material for: The Prospects of Using Structural Phase Analysis of Microcalcifications in Breast Cancer Diagnostics
Source: Diagnostics (Basel). 2023 Feb 15;13(4):737. doi: 10.3390/diagnostics13040737 (PMC9955541; doi:10.3390/diagnostics13040737)
Supplement: Supplementary file 1 [file diagnostics-13-00737-s001.zip › diagnostics-2153965-Supplementary.pdf]

diagnostics

MDPI

Table S1. Group I—The list of patients with breast cancer microcalcificates.

Case	Age of Women	Stage	TNM			Grade	Phase
			T	N	M		
**1**	43	II	2	1	0	2	HAP
**2**	63	II	2	0	0	2	HAP
**3**	62	IIA	2	1	0	2	HAP
**4**	50	II	2	1	0	2	HAP
**5**	76	II	2	1	0	3	HAP
**6**	60	II	2	0	0	2	HAP
**7**	78	II	2	0	0	2	HAP, oxalate
**8**	77	I	1	0	0	1	HAP
**9**	62	II	2	1	0	2	HAP
**10**	71	II	2	0	0	2	HAP
**11**	59	I	1	0	0	1	HAP, oxalate
**12**	51	IIA	2	0	0	2	HAP
**13**	76	IIB	2	1	0	2	HAP
**14**	64	II	2	0	0	1	HAP
**15**	65	IIB	2	1	0	2	HAP
**16**	69	I	1	0	0	3	HAP
**17**	50	IIB	2	1	0	3	HAP
**18**	66	IIB	2	1	0	1	HAP, oxalate
**19**	62	IIA	2	0	0	1	HAP
**20**	61	IIA	2	0	0	3	HAP
**21**	57	IIA	2	0	0	3	HAP, oxalate
**22**	58	IIB	2	2	0	3	HAP, oxalate
**23**	60	IIA	2	0	0	3	HAP
**24**	75	IIA	2	0	0	3	HAP
**25**	72	IIB	2	1	0	2	HAP, oxalate
**26**	47	IIB	2	1	0	2	HAP
**27**	76	IIA	2	0	0	3	HAP
**28**	55	IIB	2	1	0	2	HAP
**29**	74	IIB	2	1	0	2	HAP
**30**	64	IIA	2	1	0	2	HAP

Table S2. Group II—Breast cancer patients without calcificates.

Case	Age of Women	Stage	TNM			Grade
			T	N	M	
**1**	60	IIA	2	0	0	2
**2**	57	II	1	0	0	2
**3**	63	IIA	1	1	0	2
**4**	60	IIA	1	1	0	2
**5**	75	II	1	0	0	2
**6**	54	IIB	2	1	0	2
**7**	59	II	2	0	0	3
**8**	54	III	4	1	0	2
**9**	52	I	1	0	0	2
**10**	61	IIB	2	1	0	2
**11**	52	IIA	2	0	0	2
**12**	50	II	1	1	0	2
**13**	54	I	1	0	0	1
**14**	63	II	2	0	0	2
**15**	63	IIA	2	1	0	2
**16**	59	II	1	0	0	2
**17**	61	IIB	1	1	0	3
**18**	67	IIA	2	0	0	3
**19**	67	IIA	2	0	0	1
**20**	67	III	4	1	0	3
**21**	66	II	2	0	0	3
**22**	78	IIB	2	1	0	3
**23**	66	IIA	2	0	0	3
**24**	67	IIB	2	1	0	1
**25**	79	IIB	2	1	0	3
**26**	57	IIB	2	1	0	3
**27**	78	IIA	2	0	0	2
**28**	49	II	2	0	0	1
**29**	53	IIB	2	1	0	2
**30**	62	II	2	0	0	2
